# High Prevalence of Metabolic Syndrome in Patients with Discoid Lupus Erythematosus: A Cross-Sectional, Case-Control Study

**DOI:** 10.1155/2017/3972706

**Published:** 2017-01-03

**Authors:** Sevgi Akarsu, Ozlem Ozbagcivan, Fatma Semiz, Sebnem Aktan

**Affiliations:** Department of Dermatology, Dokuz Eylul University, Faculty of Medicine, Izmir, Turkey

## Abstract

Although it is known that systemic form of lupus erythematosus (LE) and metabolic syndrome (MetS) are frequently observed together, there are no published reports on MetS in patients with skin-restricted LE. We aimed to compare the frequencies of MetS and its components in discoid LE (DLE) with the non-DLE control group. Additionally, we intended to determine the differences of sociodemographic and clinical data of the DLE patients with MetS compared to the patients without MetS. This was a cross-sectional, case-control study, including 60 patients with DLE and 82 age- and gender-matched control subjects. In DLE group, the presence of MetS was observed as more frequent (48.3% versus 24.4%, *p* = 0.003), and hypertriglyceridemia (43.3% versus 22.0%, *p* = 0.006) and reduced HDL-cholesterol (61.7% versus 23.2%, *p* < 0.001) among the MetS components were found significantly higher when compared to the control group. DLE patients with MetS were at older age (50.45 ± 11.49 versus 43.06 ± 12.09, *p* = 0.02), and hypertension, hyperlipidemia/dyslipidemia, and cardiovascular disease histories were observed at a higher ratio when compared to the patients without MetS. Between the DLE patients with and without MetS, no significant difference was observed in terms of clinical characteristics of DLE. Moreover, further large case-control studies with follow-up periods would be required to clearly assess the impact of MetS on the clinical outcomes of DLE.

## 1. Introduction

Lupus erythematosus (LE) is a chronic relapsing immune-mediated inflammatory disease, characterized by a wide spectrum of presenting symptoms which range from a localized cutaneous form (cutaneous LE, CLE) to a life-threatening systemic form (systemic LE, SLE). CLE, defined as isolated cutaneous lupus lesions occurring in the absence of significant evidence of SLE, is two to three times more frequent than SLE. Chronic discoid LE (DLE) is the most common form of CLE. DLE occurs more frequently in women in their fourth and fifth decades of life, which appears to be well-demarcated, erythematous, scaly, keratotic plaques and eventually leading to disfiguring scarring and skin atrophy. Patients with DLE generally have a more benign disease course as compared to patients with other CLE subtypes, with only a reported 5–10% developing SLE throughout their disease course [1–3].

DLE is a multifactorial disease that involves a complex interplay between genetics and environmental triggers in the setting of adaptive and innate immune response. The mechanism has not been fully elucidated, but increasing evidence suggests the role of ultraviolet irradiation, immune dysregulation, aberrant cell signaling pathways through the cytokine cascades (T helper-1 mediators including IFN-*α*, IL-1, IL-6, IL-8, IL-10, and TNF-*α*), apoptosis, necrosis, autoantibodies, plasmacytoid dendritic cells, T cells, B cells, and vascular changes [1–3]. Although SLE and DLE are clinically different diseases, there are multiple genetic similarities between the two groups to consider a common pathogenesis [2, 3]. Although inflammatory load is lower in the patients with LE having only skin involvement; when pathogenesis is considered, it can be thought that some comorbidities accompanying SLE can also be seen in CLE patients. Hence, in the recent studies, it has been shown that some disorders such as cardiovascular diseases (CVD) and cerebrovascular accidents that are mostly associated with SLE are higher in CLE patients when compared to the control group [4–6]. Furthermore, it has been determined that metabolic syndrome (MetS) components, such as central obesity, hypertension (HT), dyslipidemia, and glucose intolerance that are known to be the risk factors in the occurrence of such diseases, are frequent in SLE patients and the presence of MetS accompanying SLE has been found to have higher ratios (varying from 17% to 40%) when compared to the general population [7–16]. Although the association of SLE and MetS has been known, no study examining the frequency of MetS in the patients with skin-restricted lupus is found in the literature.

In this study, we aimed to determine the existing MetS components and the presence of MetS in DLE patients and to compare their frequencies with the control group. In addition, we intended to describe the differences of DLE patients with MetS and the patients without MetS in terms of sociodemographic data, clinical findings, and disease severity scores.

## 2. Materials and Methods 

### 2.1. Study Design

This was a cross-sectional, case-control study, including 60 patients with DLE and 82 age- and gender-matched control subjects who have consecutively admitted to the Department of Dermatology of Dokuz Eylul University Faculty of Medicine in Izmir, Turkey, between February 2015 and June 2016. The study protocol was approved by the Local Ethical Committee which follows the guidelines set by the Declaration of Helsinki. Only DLE-diagnosed patients who had not meet SLE classification criteria were enrolled in this study. Diagnosis of DLE was based on clinicopathological presentation and additional laboratory data, if necessary. The control group included subjects without any chronic inflammatory skin disease. Exclusion criteria were <18 years old, pregnancy, and cases who had received drugs in the past that alter the metabolic parameters such as systemic corticosteroids, retinoid acid, cyclosporine, and methotrexate. Each participant gave a written informed consent prior to the examinations. After signing the informed consent, sociodemographic findings (age, gender, and smoking habits) and medical histories including diabetes mellitus (DM) and/or antidiabetic use, HT and/or antihypertensive use, hyperlipidemia/dyslipidemia and/or antihyperlipidemic use, CVD (coronary artery disease including angina, myocardial infarction, coronary bypass and stroke, peripheral arterial disease, and heart failure), and depression and/or anxiety of all the patients and the control cases were recorded. A basic psychiatric interview was requested from the psychiatric department for patients who described depression and/or anxiety and/or antidepressant and/or anxiolytic drug use to confirm the diagnosis. But standard questionnaires were not used to determine the diagnosis and severity of depression and anxiety.

We considered that current smokers were those who were smoking at the time of the interview and had been smoking at least 4 cigarettes/day for 4 years [17]. In DLE patients, disease-specific findings such as disease duration, history of daily use of antimalarial treatment for more than 3 months, location and distribution of the lesions, and Cutaneous Lupus Erythematosus Disease Area and Severity Index (CLASI) scores were also recorded. Then, laboratory examinations and biometric measurements were performed both in DLE patients and in the control group in terms of dyslipidemia and MetS components, and the presence of MetS was investigated.

### 2.2. Clinical Severity Measurements in DLE Patients

In all DLE patients, severity of lesions was evaluated according to the CLASI which consists of activity and damage scores. This tool considers the anatomical location and morphology of the lesions and has been validated for use by both dermatologists and rheumatologists [18, 19]. The activity score considers erythema (0–3), scale/hypertrophy (0–2), mucous membrane lesions (0-1), recent hair loss (0-1), and nonscarring alopecia (0–3), and the damage score considers dyspigmentation (0-1), scarring/atrophy/panniculitis (0–2), and scarring of the scalp (0–6). Patients were asked for their dyspigmentation durations and the dyspigmentation score was doubled if it lasted for 12 months or longer [19].

### 2.3. Biometric and Laboratory Screenings

Body mass index (BMI) was calculated using the formula weight (kg)/height (m)^2^. Waist circumference (cm) was measured along the line lying midway between the iliac crest and the costal margin in the midaxillary line. Blood pressure (BP) was obtained using the validated device at our center and recordings were made for two times with 1-minute intervals, and the average of both values was taken as the true BP of the patients. Blood samples were taken after an overnight fasting of 12 h and levels of glucose (fasting blood glucose, FBG), total cholesterol (TC), triglycerides (TG), high-density lipoprotein cholesterol (HDL-C), and low-density lipoprotein cholesterol (LDL-C) were measured. The presence of dyslipidemia was defined as serum TG >150 mg/dL, TC >200 mg/dL, LDL-C >130 mg/dL, and/or antihyperlipidemic treatment [20]. When calculating the mean values of TC, TG, LDL-C, HDL-C, FBG, and systolic BP/diastolic BP, the values of the patients using antihyperlipidemic treatment, hypoglycemic treatment, and antihypertensive treatment were excluded from the calculation because of the fact that they will not reflect the actual values.

### 2.4. Assessment of MetS

The presence of MetS at study visit was assessed in line with the consensus criteria which were recently approved by Alberti et al. as a joint interim statement of the International Diabetes Federation Task Force on Epidemiology and Prevention; National Heart, Lung, and Blood Institute; American Heart Association; World Heart Federation; International Atherosclerosis Society; and International Association for the Study of Obesity [21]. Patients were accepted to have MetS if three or more of the following criteria were present: increased waist circumference (≥80 cm for female, ≥94 cm for male), increased TG (≥150 mg/dL or drug treatment for hypertriglyceridemia), reduced HDL-C (<50 mg/dL for female, <40 mg/dL for male, or drug treatment for reduced HDL-C), arterial HT (systolic ≥ 130 and/or diastolic ≥85 mm Hg or drug treatment for HT), and increased FBG (≥100 mg/dL or drug treatment for DM) [21, 22].

### 2.5. Statistical Analyses

The statistical analyses were performed with the SPSS/PC software (Version 22.0 for Windows; SPSS Inc., Chicago, IL). Constant variables in the data set were expressed as mean ± standard deviation, and categorical variables were expressed as frequency and percentage. The Shapiro-Wilk test was used to examine the normality of the distribution of the data. Two samples' Student's *t*-test was used to compare mean values of normally distributed quantitative variables as the two samples were obtained independently. Mann–Whitney *U* test was used if the variables were not normally distributed. Qualitative variables were analyzed with chi-squared test and Fisher's exact test. *p* < 0.05 was considered significant in all analyses.

## 3. Results

The average age of 60 DLE patients included in this age- and gender-matched controlled study was 46.63 ± 12.28 years old and the gender of majority was female (female/male: 2.33). In DLE group, it was determined that the presence of MetS was more frequent than the control group (48.3% versus 24.4%, *p* = 0.003), and, among the MetS components, only hypertriglyceridemia (43.3% versus 22.0%, *p* = 0.006) and reduced HDL-C (61.7% versus 23.2%, *p* < 0.001) were found to be significantly higher. Additionally, being current smokers, depression/anxiety history, and presence of dyslipidemia were significantly higher in DLE patients than the control group (Table 1). Five (8.3%) of DLE patients (2 (3.3%) had angina, 2 (3.3%) had myocardial infarction, and 1 (1.7%) had coronary bypass) and 4 (4.9%) of controls (3 (3.6%) had angina and 1 (1.2%) had myocardial infarction) had coronary artery disease in means of CVD.

When DLE patients with and without MetS were compared with each other, it was determined that the patients with MetS did not show any difference in terms of gender distribution when compared to the patients without MetS; however they were at older age (50.45 ± 11.49 versus 43.06 ± 12.09, *p* = 0.02). In addition, while no statistically significant difference was found between both groups in terms of smoking status and mean BMI values, HT and/or antihypertensive use, hyperlipidemia/dyslipidemia and/or antihyperlipidemic use, and CVD histories were found higher in DLE patients with MetS. In DLE patients with MetS, the frequencies of MetS components were found to have increased waist circumference (86.2%), reduced HDL-C (79.3%), hypertriglyceridemia (72.4%), elevated BP (62.1%), and increased FBG (58.6%), respectively. As expected, all individual components of MetS were more common in patients with MetS than in those without (Table 2).

It was determined that the prevalence of CVD was increasing by age in a statistical significance in DLE patients, as well as the prevalence of MetS (*p* = 0.001). DLE patients with MetS who had CVD had statistically significant higher mean age (64.00 ± 9.46) compared to those without CVD (45.00 ± 11.29) (*p* = 0.002). In partial correlation analysis, ignoring the age factor, the association between MetS and CVD did not reach a statistical significance (*p* = 0.109).

No statistically significant difference was observed between the DLE patients with and without MetS in terms of DLE disease duration, use of antimalarial treatment, lesion locations, distribution of lesions, and the mean total CLASI activity score, damage score, and subscale scores (Table 3).

## 4. Discussion

In this study, we have determined the prevalence of MetS and its individual components in a sample of DLE patients from Turkey. As a result, we found higher rates of MetS in DLE patients (48.3%) than the control group as well as lower values of HDL-C (61.7%) and higher values of triglycerides (43.3%) among the MetS components. In DLE patients with MetS, histories of hyperlipidemia/dyslipidemia, HT, and CVD were notified more frequent, and the presence of dyslipidemia and all MetS components was observed at a significantly higher ratio than the patients without MetS through the investigations.

MetS is characterized by a group of interrelated metabolic risk factors, including the central obesity, atherogenic dyslipidemia, elevated BP, and increased FBG. It is known that these risk factors promote the development of atherosclerotic CVD and/or type 2 DM. The prevalence of MetS has increased in the last few years throughout the world. Approximately, one-third of the adult population in industrialized and also developing countries can be categorized as having MetS by different definitions [22, 23]. Recent evidences suggest that SLE has been associated with MetS as well as its various components [7–16]. The most important reason of the long-term mortality in SLE is premature atherosclerosis and CVD and, in such patients, it has been observed that both MetS and MetS components have higher prevalence and also cumulative organ damage correlated with SLE in the presence of MetS is higher [10–15]. Some authors have mentioned that whether lupus activity is a factor in the development of MetS or whether MetS is contributing to disease activity is unclear. Inflammation is not only a triggering factor of the MetS but also a consequence. It is thought that increased circulating adipocytokines such as TNF-*α*, IL-6, leptin, resistin, plasminogen activator inhibitor-1, and acute-phase reactants such as C-reactive protein could have an important role in the development of MetS and/or its components and its association with disease activity in SLE patients [12–15].

As known, a critical and most important part of assessment of DLE patients is evaluating and monitoring for early detection of SLE development [1–3]. Furthermore, especially in recent years, evaluating the significant accompanying comorbidities in terms of morbidity and mortality such as CVDs and cerebrovascular accidents (which are more frequent in SLE) has been highlighted in skin-restricted lupus patients [4–6]. However, it is unknown that whether CLE is associated with risk of MetS or not. To date, there are no published reports on MetS and associated factors in patients with CLE.

Recently, in a large case-control study performed by Hesselvig et al. in Danish population, significantly increased risk of CVDs and all-cause mortality were observed both in CLE and in SLE patients including inpatients and outpatients, as being higher in SLE, and these risks were found more significant in the patients with an age of ≤50 years [4]. In a Swedish study, Zöller et al. examined whether there was an association between hospital admission for CLE and subsequent risk of hospitalization for coronary heart disease. They found that the risk was at a greatest level 1–5 years after being hospitalized with CLE and age of >50 years with a higher standardized incidence ratio for subsequent coronary heart disease of patients with DLE after one year of follow-up [5]. Furthermore, in a population-based inception cohort of patients with CLE, while the cardiovascular death, ischemic heart disease, and heart failure risks were similar to the control group, cerebrovascular accidents and peripheral arterial disease risks were higher at a ratio of at least two times. Although these risks of cardiovascular events and cerebrovascular accidents did not show a statistical significance in generalized DLE, it was specified that such risks were higher than the patients with localized involvement; thus these comorbidities could increase depending on the extent of cutaneous involvement [6]. In our study, the presence of CVD history did not show significance in DLE patients when compared to the control group; however, it was observed higher in DLE patients with MetS when compared to the patients without MetS. Moreover, because of the absence of a significant relationship between CVD and MetS when the age factor was ignored, it can be said that the combination of CVD and MetS increases by age in DLE patients. The MetS is not an absolute risk indicator because it does not contain many of the factors that determine absolute risk, for example, age, sex, cigarette smoking, and LDL-C levels. Nonetheless, patients with the MetS are at twice the risk of developing CVD over the next 5 to 10 years as individuals without the syndrome. The risk over a lifetime undoubtedly is even higher [9–13, 20]. Similarly, the ratio of generalized lesion distribution, the mean total CLASI activity, and damage scores were higher in our DLE patients with MetS than in those without MetS, but such values did not reach a statistical significance. The notion that antimalarials are protective against MetS, CVD, and general mortality in SLE patients is endorsed by some authors [13, 15], but not by others [6, 14]. Singh et al. found that there was no difference in total cholesterol between CLE patients who had or never used antimalarials [6]. Additionally, the use of antimalarial therapy in DLE patients did not show an association with MetS in our study. Thus, more long-term results from prospective large cohorts would be needed to address these arguments.

The pathogenesis of CLE is similar to SLE, with complex gene-environment interactions, autoimmunity, and immune-mediated cutaneous damage, and, hence, conceivably, patients with CLE may have increased risk of MetS. Local and chronic inflammation in DLE patients may be the driver of low-grade systemic inflammation [2–4]. Thus, this inflammation may have thought of to be the connecting link between DLE and MetS. Although the limited extent of organ involvement with absence of systemic damage suggests that chronic systemic inflammation may be absent in patients with CLE, other limited autoimmune dermatological conditions such as psoriasis, alopecia, and lichen planus have also been variably associated with an increased MetS and/or its components [24].

In this study, the most frequently determined MetS components in DLE patients were increased waist circumference, reduced HDL-C, and hypertriglyceridemia conforming to the previous findings of SLE patients with MetS. Unsurprisingly, it was an expected result to find lower ratio of arterial HT in DLE patients with MetS when compared to the SLE patients with MetS. In the previous studies, older age in SLE patients has been notified with MetS similar to our DLE patients; however, different from our study, as can be interpreted as higher inflammation burden in SLE, higher BMI, longer disease duration, and higher disease activity have been found in SLE patients with MetS when compared to the patients without MetS [13, 14].

Several studies have yielded crucial information implicating visceral adipose tissue as a main focus of the chronic inflammation that can contribute to MetS and its components. It has been demonstrated that the downregulation of T-regulatory cells seems to play an important role in the development of CLE and these T-regulatory cells also ameliorate the glucose intolerance in the context of central obesity. Additionally, it has been shown that B cells exacerbate glucose intolerance through the production of pathogenic immunoglobulins presumably against adipose tissue antigens [16]. In our study, the presence of increased FBG in DLE patients did not show any significance when compared to the control group, and it can be attributed to the comparable values of the indicators of visceral adipose tissue such as mean BMI and mean waist circumference in patients and control group. Also, both increased waist circumference and increased FBG frequencies were higher in the DLE patients with MetS when compared to the DLE patients without MetS.

DLE has been linked to smoking in several case-control studies, which have shown to have consistently higher smoking rates (50–87%) in DLE patients [17, 25, 26]. It has been shown that cigarette smoking increases inflammatory cytokines, apoptosis, certain autoantibodies, the development of free radicals, and photosensitivity [17]. We found a statistically higher number of smokers among DLE patients than among controls and the number of cigarettes smoked daily was higher in patients with DLE, as found in other case-control reports. In previous studies, it has been determined by Negrón et al. that smoking status in SLE patients with MetS did not show any significance when compared to the control group while Mederios et al. reported that SLE patients with MetS were higher smokers when compared to the patients without MetS [12, 13]. In our study, no significance was observed in terms of smoking habits between the DLE patients with and without MetS.

In recent years, the common association of skin-restricted LE with psychiatric disease and daily stress has been demonstrated in a few studies [27–30]. In a case-control study conducted by Jalenques et al., it was found that the most frequently observed current psychiatric disorders were major depressive disease (39%) and anxiety disorders (35%) in patients with skin-restricted lupus [28]. In another cross-sectional study, 34.7% of the patients with DLE met the criteria for depression and/or anxiety with need of mental health care or psychotropic treatment [29]. Skin atrophy, scarring, and scarring alopecia may cause mood and anxiety disorders and can seriously impact the quality of life in patients with DLE. Additionally, anxiety and emotional factors can also precipitate DLE lesions and induce treatment resistance [28–30]. It has been suggested that chronic inflammation may be a key feature linking DLE with depression and/or anxiety. Also it has been notified that the presence of depression can be related to both SLE and MetS and/or its components via common pathogenesis. In other words, depression is a contributing factor to increased (neuro)inflammatory burden; therefore it may increase the inflammatory and degenerative progression. It is now evident that depression is the clinical expression of peripheral cell-mediated activation, inflammation (increased levels of proinflammatory cytokines, such as IL-6 and TNF-*α*), and induction of oxidative and nitrosative stress pathways [30]. In our study, depression and/or anxiety ratio was significantly found to be higher in DLE patients when compared to the control group; however, although it was partially higher in DLE patients with MetS when compared to the patients without MetS, no statistically significance was observed.

Consequently, when the MetS frequency and the number of determined MetS components in DLE patients were compared to the non-DLE control group, they were found to be approximately two times higher. All the components in DLE patients with MetS were more frequent than in the patients without MetS and they were central obesity, reduced HDL-C, hypertriglyceridemia, HT, and glucose intolerance, respectively. In these patients, CVD history was also determined more frequently. Due to the absence of data in the literature related to the presence of MetS in skin-restricted LE patients, these results obtained from our study can be a fore step for further studies. Nevertheless, the present study has some limitations such as being a cross-sectional design and including small number of patients with DLE. Additionally, other factors known to be associated with MetS, particularly alcohol drinking, dietary and exercise habits, and proinflammatory cytokines or procoagulant factors such as TNF-*α*, IL-6, and fibrinogen, were not evaluated in this study.

## 5. Conclusions

In conclusion, the frequencies of MetS and especially the MetS components correlated with atherogenic dyslipidemia, which are independent risk factors for atherosclerotic CVD, were found to be at a high ratio in DLE. Thus, it may be said that it is important to identify patients at risk to develop MetS early in the course of DLE. In such patients, in terms of reducing the risk of MetS, applying lifestyle changing strategies such as nutritional counselling and weight reduction can be recommended. Because of its design, our study does not indicate cause and effect between DLE and MetS and its components, but inflammation might be an area with potential for further investigation. Therefore, further large case-control studies with follow-up periods would be required to clearly assess the impact of MetS on the clinical outcomes of DLE.

## Figures and Tables

**Table 1:** Comparison of sociodemographic features, medical history findings, and metabolic syndrome components in study subjects.

Characteristics	DLE (*n* = 60)	Controls (*n* = 82)	*p* value
Age, years, mean ± SD	46.63 ± 12.28	46.40 ± 12.00	0.911
Gender, *n* (%)			
Male	18 (30)	25 (30.5)	0.950
Female	42 (70)	57 (69.5)	
Current smokers, *n* (%)	34 (56.7)	26 (31.7)	0.003^*∗*^
Packet/year	16.53 ± 14.73	4.02 ± 8.26	<0.001^*∗*^
Medical history, *n* (%)			
Diabetes mellitus and/or antidiabetic use	6 (10)	9 (11)	0.852
Hypertension and/or antihypertensive use	18 (30)	14 (17.1)	0.069
Hyperlipidemia/dyslipidemia and/or antihyperlipidemic use	4 (6.7)	1 (1.2)	0.162
Cardiovascular disease	5 (8.3)	4 (4.9)	0.494
Depression and/or anxiety	24 (40)	7 (8.5)	<0.001^*∗*^
Body mass index, mean ± SD	27.08 ± 3.90	26.67 ± 4.95	0.385
Dyslipidemia (%)	40 (66.7)	38 (46.3)	0.016^*∗*^
Metabolic syndrome	29 (48.3)	20 (24.4)	0.003^*∗*^
Increased waist circumference ≥ 94 cm (male) or ≥80 cm (female)	38 (63.3)	49 (59.8)	0.666
Hypertriglyceridemia ≥ 150 mg/dL or antihyperlipidemic treatment	26 (43.3)	18 (22.0)	0.006^*∗*^
Reduced HDL-cholesterol < 50 mg/dL (female) or <40 mg/dL (male)	37 (61.7)	19 (23.2)	<0.001^*∗*^
Elevated blood pressure ≥ 130/85 mmHg or antihypertensive treatment	25 (41.7)	26 (31.7)	0.222
Increased fasting blood glucose ≥ 100 mg/dL or hypoglycemic treatment	20 (33.3)	22 (26.8)	0.402
Number of metabolic syndrome components	2.37 ± 1.33	1.48 ± 1.27	<0.001^*∗*^

^*∗*^Statistically significant values.

**Table 2:** Comparison of sociodemographic features, medical history findings, and metabolic syndrome components in DLE patients with and without metabolic syndrome.

Characteristics, *n* (%)	DLE patients with MetS (*n* = 29)	DLE patients without MetS (*n* = 31)	*p* value
Age, years, mean ± SD	50.45 ± 11.49	43.06 ± 12.09	0.020^*∗*^
Gender, *n* (%)			
Male	11 (37.9)	7 (22.6)	0.195
Female	18 (62.1)	24 (77.4)	
Current smokers, *n* (%)	17 (58.6)	17 (54.8)	0.768
Packet/year	16.86 ± 17.10	16.22 ± 12.42	0.715
Medical history, *n* (%)			
Diabetes mellitus and/or antidiabetic use	5 (17.2)	1 (3.2)	0.098
Hypertension and/or antihypertensive use	14 (48.3)	4 (12.9)	0.003^*∗*^
Hyperlipidemia/dyslipidemia and/or antihyperlipidemic use	4 (13.8)	0	0.049^*∗*^
Cardiovascular disease	5 (17.2)	0	0.022^*∗*^
Depression and/or anxiety	12 (41.4)	12 (38.7)	0.833
Body mass index, mean ± SD	28.01 ± 3.74	26.22 ± 391	0.096
Dyslipidemia, *n* (%)	23 (79.3)	17 (54.8)	0.044^*∗*^
Metabolic syndrome components			
Increased waist circumference ≥ 94 cm (male) or ≥ 80 cm (female)	25 (86.2)	13 (41.9)	<0.001^*∗*^
Hypertriglyceridemia ≥ 150 mg/dL or antihyperlipidemic treatment	21 (72.4)	5 (16.1)	<0.001^*∗*^
Reduced HDL-cholesterol < 50 mg/dL (female) or <40 mg/dL (male)	23 (79.3)	14 (45.2)	0.007^*∗*^
Elevated blood pressure ≥ 130/85 mmHg or antihypertensive treatment	18 (62.1)	7 (22.6)	0.002^*∗*^
Increased fasting blood glucose ≥ 100 mg/dL or hypoglycemic treatment	17 (58.6)	3 (9.7)	**<0.001**

^*∗*^Statistically significant values.

**Table 3:** Comparison of clinical characteristics in DLE patients with and without metabolic syndrome.

Characteristics, *n* (%)	DLE patients with MetS (*n* = 29)	DLE patients without MetS (*n* = 31)	*p* value
Disease duration (month), mean ± SD	74.10 ± 61.05	68.52 ± 75.36	0.210
Use of antimalarials	8 (27.6%)	9 (29)	0.901
Location of DLE lesions, *n* (%)			
Face	25 (86.2)	27 (87.1)	1.00
Scalp	12 (41.4)	11 (35.5)	0.639
Trunk	9 (31)	8 (25.8)	0.653
Upper extremity	8 (27.6)	9 (29)	0.901
Lower extremity	2 (6.9)	2 (6.5)	1.00
Distribution of DLE lesions, *n* (%)			
Localized	18 (62.1)	21 (67.7)	0.645
Generalized	11 (37.9)	10 (32.3)	0.645
CLASI scores, mean ± SD			
Total CLASI activity score	9.10 ± 9.03	7.32 ± 6.42	0.588
Erythema	6.00 ± 6.24	4.58 ± 4.23	0.346
Squama	2.34 ± 3.40	1.90 ± 2.20	0.939
Mucosal involvement	0.21 ± 0.41	0.23 ± 0.42	0.860
Noncicatricial alopecia	0.76 ± 0.99	0.71 ± 1.01	0.777
Total CLASI damage score	5.93 ± 6.22	4.55 ± 5.36	0.292
Dyspigmentation	2.28 ± 2.53	1.58 ± 2.25	0.145
Scarring	1.79 ± 2.74	1.74 ± 1.97	0.562
Cicatricial alopecia	1.86 ± 2.34	1.26 ± 1.82	0.430
